# Impact of Visceral and Hepatic Fat on Cardiometabolic Health

**DOI:** 10.1007/s11886-024-02127-1

**Published:** 2024-09-05

**Authors:** Tasveer Khawaja, Matthew Nied, Abigail Wilgor, Ian J. Neeland

**Affiliations:** 1 Harrington Heart and Vascular Institute, University Hospitals Cleveland and Case Western Reserve University, Cleveland, OH USA; 2 Department of Medicine, University Hospitals Cleveland and Case Western Reserve University, 11100 Euclid Ave. Mailstop Lakeside, Cleveland, OH USA

**Keywords:** Cardiometabolic risk, Visceral fat, Hepatic fat, Obesity, SGLT2i, GLP-1 RA

## Abstract

**Purpose of Review:**

Body fat distribution plays a significant role in the cardiometabolic consequences of obesity. We review the impact of visceral and hepatic fat and highlight important interventions.

**Recent Findings:**

Several epidemiologic studies have established a clear association between visceral fat and cardiovascular disease. The association between hepatic fat and cardiovascular disease is less clear with discordant results. Novel evidence demonstrates sodium glucose co-transporter-2 (SGLT2) inhibitors facilitate modest weight loss and reductions in ectopic fat depots in patient with type 2 diabetes. Glucagon-like peptide-1 (GLP-1) receptor agonists have been associated with decreased visceral/hepatic fat and reductions in MACE in populations with type 2 diabetes and with overweight/obesity.

**Summary:**

Clear associations between visceral fat and cardiometabolic outcomes have been established, whereas the impact of hepatic fat remains less clear. Lifestyle modification and pharmacologic interventions remain the initial therapies, while surgical intervention is associated with improved long-term outcomes. Emerging therapies have demonstrated a profound impact on body fat distribution and cardiometabolic risk.

## Introduction

**Fig. 1:**
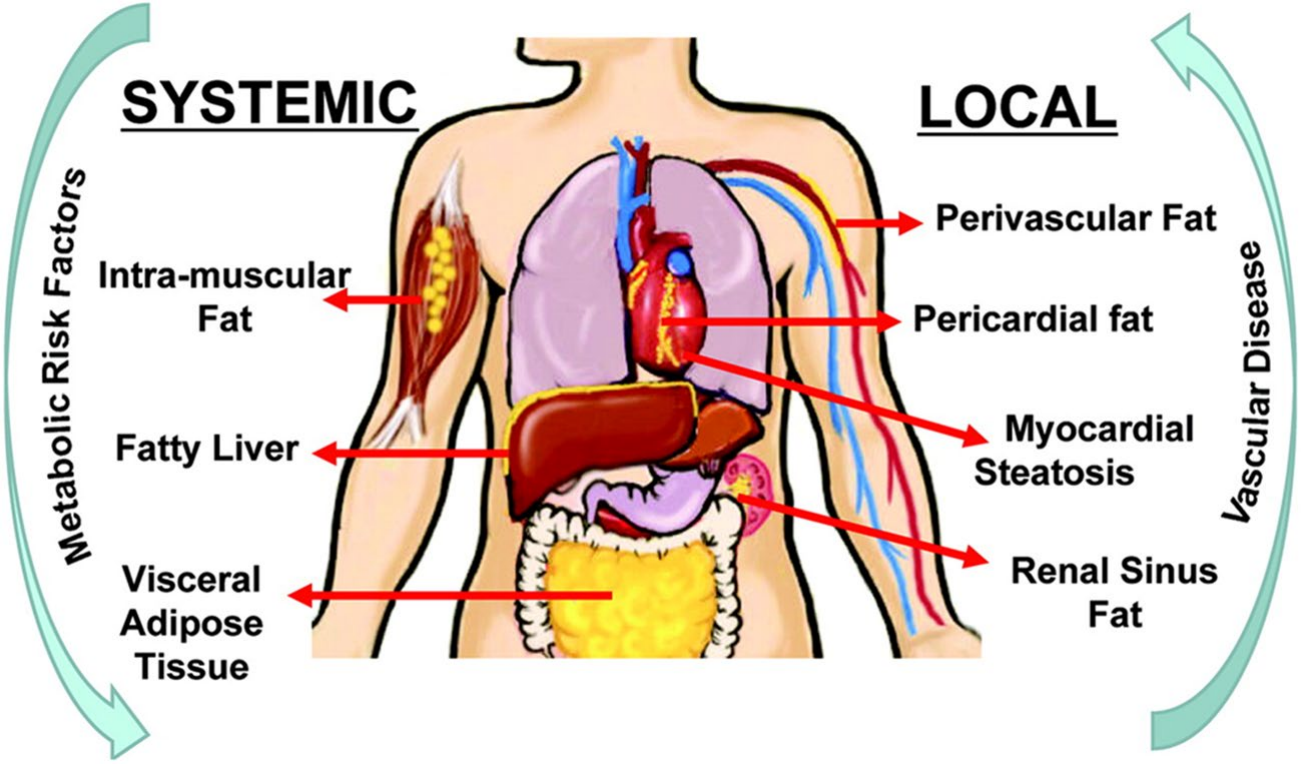
Ectopic fat depots and their potential systemic and local effects. (Figure reproduced with permission from Britton KA, Fox CS. Circulation. 2011;124:e837–41, with permission of Wolters Kluwer Health, Inc.) [1]

Obesity is a known risk factor for cardiometabolic disease. Although body mass index (BMI) is an inexpensive and easily accessible metric to define and characterize obesity at a population level, it fails to account for differences in individual body composition (lean mass versus fat mass), body fat distribution, or burden of ectopic fat such as in the liver, skeletal muscle, and heart (Fig. 1). The magnitude of fat stores in persons living with obesity can be highly variable and can make up as much as 60% of total body weight [2]. For most of these individuals, greater than 80% of their fat will be stored in the subcutaneous tissue (SAT) and the remaining will be found in visceral adipose tissue (VAT) [2]. Non-modifiable factors such as age, sex, and race/ethnicity impact the distribution of VAT versus SAT [2]. VAT has been demonstrated to be the primary driver of most of the cardiometabolic consequences of obesity and should be a target for treatment to prevent the incidence and progression of the cardiovascular-kidney-metabolic (CKM) syndrome [3, 4].

Hepatic fat is another important ectopic fat depot and has a complex relationship with cardiometabolic health. Metabolic dysfunction associated steatotic liver disease (MASLD) is a common disorder that impacts greater than 25% of adults [5]. In some subgroups, such as those with type 2 diabetes (T2D), the prevalence of MASLD approaches 50% [5]. Because of the close association of MASLD with established risk factors for cardiovascular disease (CVD), it is challenging to isolate the impact of MASLD on CVD risk. Although the leading cause of mortality in those with MASLD is CVD, the association between MASLD and CVD has been called into question. This review will serve to highlight the impact of visceral and hepatic fat on cardiometabolic outcomes, while also highlighting interventions targeted at VAT and MASLD.

### Visceral Fat

VAT and SAT differ in their metabolic activity and can lead to alterations in cardiometabolic function based on an individual’s body composition. VAT is more vascular, innervated, composed of larger, hypertrophic adipocytes, and contains more inflammatory and immune cells than SAT [6]. VAT also has higher metabolic activity as it has greater sensitivity to adrenergic stimulation and a higher capacity for free fatty acid generation and glucose uptake than SAT [6]. Because VAT is more sensitive to lipolysis and more insulin-resistant than SAT, this can have impacts on overall insulin resistance and the development of cardiovascular conditions, diabetes, and obstructive sleep apnea (OSA). Although VAT quantification may be useful in establishing the risk of obesity-related conditions, direct quantification is limited by financial, environmental, and technical constraints as it can only be measured by imaging modalities. Other indices for VAT, like the visceral adiposity index (VAI), may be used as a surrogate when imaging is not feasible. VAI can be calculated with a gender-specific algorithm that includes waist circumference, BMI, serum triglycerides, and high density lipoprotein [7]. Although VAI may be a better estimation of risk than BMI, it still fails to outline the nuance of an individual’s muscle, SAT, and VAT composition that is gained from imaging.

#### Cardiovascular Disease

The Dallas Heart Study linked visceral obesity to coronary artery disease (CAD), heart failure with preserved ejection fraction, atrial fibrillation, and hypertension [8]. The underlying mechanisms of these findings are likely multifactorial. Visceral fat can increase mechanical loads on the heart by increasing both preload and afterload. Preload is increased by higher total blood volume whereas afterload is increased by sympathetic overactivation from increased oxygen demands and low-grade inflammation from adipocyte hypertrophy [9]. Higher levels of ectopic fat distribution that occur in visceral obesity can also lead to increases in epicardial adipose tissue. Epicardial adipose tissue makes up 20% of the total ventricular weight of the heart and protects 80% of the heart’s surface [10]. During periods of high energy demand, it can transport and buffer fatty acids to be used as local energy sources [10]. This tissue is more susceptible to pro-inflammatory properties that can lead to reduced coronary blood flow from vasoconstriction [8, 11]. Subsequent diastolic dysfunction and conduction heterogeneity can predispose obese patients to higher rates of heart failure and conduction disease like atrial fibrillation [8, 11].

Recent research has focused on better understanding and quantifying VAT and CVD risk. Karlsberg et al. investigated the link between severity of VAT and CAD through coronary CTA. These findings showed there was progression of median coronary plaque volumes for every increasing quartile of VAT [12]. This correlation was seen in both low-density and other non-calcified plaque but interestingly was not shown in calcified plaque [12]. Data from the National Health and Nutrition Examination Survey (NHANES) was investigated by Huang et al. and showed that individuals with higher visceral obesity had greater arterial stiffness than their normal visceral fat counterpart [13]. This increased arterial stiffness can lead to both higher rates of major adverse cardiovascular events (MACE) and heart failure. These findings were corroborated by their analysis showing the 10-year cardiovascular risk was significantly increased in those stratified in the visceral obesity group compared to controls [13]. This data affirms that individuals with normal weight may be at elevated risk of cardiovascular events if they have a high degree of VAT [13]. By using data from NHANES, Zhang et al. demonstrated that VAI was another correlate with heart failure and an increase in 1 unit of VAI confers a 4% higher risk for heart failure [14]. While VAT alone is a risk factor for the development of CVD, the association of VAT with other comorbidities, such as diabetes and OSA, compound the risks of cardiometabolic-associated morbidity and mortality in individuals with a high degree of visceral obesity.

#### Type 2 Diabetes

T2D is expected to reach 350 million cases by 2030 and may contribute to greater than 70% of early deaths worldwide [15]. More investigations are underway to better understand the association between visceral fat and risk of T2D. Evidence supports that higher levels of VAT and VAT accumulation over time are strongly linked to a higher incidence of T2D [16, 17]. There have been several mechanisms that are hypothesized to explain this risk. One is that there is an increased deposition of free fatty acids that are mobilized into hepatic circulation from visceral fat that increases the presence of adipocyte-associated inflammatory markers. These inflammatory markers have been linked to T2D and atherosclerosis [17]. Dysfunctional VAT is also associated with changes to extracellular matrix composition and function, increased immune cells, and adipocyte hypertrophy [15]. Adipocyte hypertrophy can lead to impaired oxygen consumption by overwhelming existing tissue vasculature, leading to ischemic tissue necrosis [18]. This necrosis induces inflammatory cytokine release and fibrosis and contributes to the perpetuation of low-grade systemic inflammation that is often present in obesity. Surrounding adipocytes are susceptible to the paracrine signaling from the inflammatory response and subsequently uptake less glucose and free fatty acids [6, 9]. Individuals with a higher VAT: SAT ratio lose the buffer that SAT provides by absorbing circulating free fatty acids and triglycerides that VAT cannot [6]. The result of this signaling pathway is higher levels of insulin resistance and lipid oxidation in those with visceral obesity.

Recent investigations into the relationship between VAT and T2D have aimed at quantifying the absolute risk increase associated with visceral obesity. One study comparing VAT volume and VAT area in patients with pre-existing pre-diabetes or T2D found that higher VAT volumes by CT volumetric analysis correlated more with insulin resistance, hepatic steatosis, and cardiometabolic risk factors than VAT area alone [19]. A meta-analysis of nine cohort studies found that for an increase of one unit of visceral fat index, there was a 42% higher risk of developing T2D [20]. This was corroborated from data collected by the NHANES study by Zheng et al. which showed a 0.15 times higher risk of T2D and prediabetes for every 1 unit of VAI [14]. Further analysis of this data by Xu et al. showed that higher VAT area was positively associated with T2D across both sexes and all races/ethnicities except for Black females [21]. Subcutaneous fat lacked this association, except for in white females [21]. Although the aforementioned studies provide additional evidence to the defined relationship between VAT and T2D, there remains an opportunity for further investigations to provide a more accessible quantification of VAT to apply to risk calculations.

#### Obstructive Sleep Apnea

Both functional and anatomic features that are commonly found in metabolic syndrome can predispose an individual to OSA. Increased mechanical loads on the pharynx can lead to pharyngeal collapse and airway obstruction during sleep. In obesity, excess fat deposition can affect the airway both directly and indirectly. Fat deposition in the pharyngeal structures of the tongue, soft palate, and uvula can have direct effects on airway patency [22]. Indirect effects of upper abdominal fat mass and recumbent posture can lead to decreased lung volumes, decreased tracheal traction forces and pharyngeal wall tension [23]. The other primary risk factor for the development of OSA is increased age. Recent research has predicted that one of the primary mechanisms for OSA development in people of advanced age is the increase in their percentage of visceral fat [24, 25]. D’Angelo et al. found that age was inversely associated with tongue and upper abdominal muscle attenuation, an indicator that older individuals had a higher degree of visceral fat [25]. In a retrospective cohort study by Sekizuka et al., when analyzing visceral fat by bioelectrical impedance analysis, they found that visceral fat area was a stronger coexisting factor for OSA than gender, age, or BMI [12]. They also found that visceral fat was the only significant component of OSA severity [26]. A large cross-sectional study bolstered data for the claims that elevated visceral fat index was significantly associated with OSA risk [27–29]. Although the treatment of choice for OSA is nocturnal CPAP, a meta-analysis of 11 studies showed that CPAP use does not reduce the amount of VAT [30]. Despite this, early identification and treatment of OSA can reduce the risk of cardiovascular comorbidities that often accompany OSA.

### Hepatic Fat

The relationship between hepatic fat and CVD is complex and nuanced. Several studies have demonstrated an association between MASLD and ASCVD (Table 1). In a recent retrospective study of greater than 100,000 participants without baseline CVD, liver disease, or cancer, fatty liver disease was diagnosed by ultrasonography during regular health screening exams. On follow up, 183 participants developed myocardial infarction (MI). After adjustment for several covariates, the presence of MASLD was significantly associated with MI (HR 1.54; 95% CI 1.11–2.14) [31]. Mechanistically, the association between liver fat and ASCVD risk is thought to be related to both an independent association between these entities, as well as an interaction between MASLD severity and other important ASCVD risk factors, including T2D and hypertension [5]. Notably, MASLD is theorized to be associated with endothelial dysfunction, heightened systemic inflammatory tone, and ectopic fat deposition in other organs [5].

**Table 1:** Summary of studies that evaluated the Association between MASLD and ASCVD Risk

Reference	Diagnostic modality	Patients, *n*	Type of study	Impact of MASLD on CVD outcomes or ASCVD compared with control subjects after adjustment for risk factor covariates
Jepsen et al., 2003	Ultrasound	1804	Retrospective	OR, 2.1 for CVD mortality
Targher et al., 2007	Ultrasound	2839	Cross-sectional	OR, 1.49 for CAD, PAD, and cerebrovascular disease in type 2 diabetes
Hamaguchi et al., 2007	Ultrasound	1637	Prospective	HR, 4.1 for nonfatal CVD events
Santos et al., 2007	Ultrasound	505	Cross-sectional	OR, 1.73 for coronary calcification
Haring et al., 2009	Ultrasound	4160	Prospective	HR, 6.22 for all-cause and CVD mortality
Assy et al., 2010	CT	61	Cross-sectional	OR, 2.03 for coronary calcification
Chen et al., 2010	Ultrasound/CT	295	Cross-sectional	OR, 2.46 for CAC > 100
Wong et al., 2011	Ultrasound	612	Prospective	OR, 2.31 for significant coronary artery disease (> 50% obstruction)
Targher et al., 2012	Ultrasound	343	Cross-sectional	OR, 7.6 for CAD, PAD, and cerebrovascular disease in type 1 diabetes
Kim et al., 2012	Ultrasound	4023	Cross-sectional	OR, 1.32 for CAC > 10
Zhou et al., 2012	Ultrasound	3543	Prospective	OR, 3.0 for CVD mortality
Stepanova and Younossi, 2012	Ultrasound	20 050	Prospective	OR, 1.23 for CVD events
Ekstedt et al., 2015	Liver biopsy	229	Retrospective	HR, 1.55 for CVD mortality
Mellinger et al., 2015	CT	3014	Cross-sectional	OR, 1.20 for CAC score > 90th percentile for age
Mantovani et al., 2016	Ultrasound	286	Retrospective	OR, 6.73 for incident cardiovascular events in type 1 diabetes
Pais et al., 2016	Fatty Liver Index	5671	Retrospective	MASLD severity correlates with CIMT and carotid plaque severity
Yoshitaka et al., 2017	Ultrasound	1647	Prospective	HR, 10.4 in nonoverweight, 3.1 in overweight for incident cardiovascular events
Mahfood Hadad et al., 2017	Ultrasound	25 837 (11 studies)	Meta-analysis	RR, 1.77 for incident CVD, 1.43 for cardiovascular mortality
Zhou et al., 2018	Ultrasound/CT	8346 (6 studies)	Meta-analysis	OR, 2.20 for incident CVD in patients with diabetes
Kapuria et al., 2018	Ultrasound/CT	42 410 (12 studies)	Meta-analysis	OR, 1.64 for higher CAC scores
Sinn et al., 2019	Ultrasound	111 492	Retrospective	HR, 1.54 for myocardial infarction
Pais et al., 2019	Fatty Liver Index	2554	Retrospective	MASLD correlated with CIMT, CAC, and carotid plaque
*ASCVD* indicates atherosclerotic cardiovascular disease, *CAC* coronary artery calcium, *CIMT* carotid intima-media thickness, *CT* computed tomography, *CVD* cardiovascular disease, *HR* hazard ratio, *MASLD* metabolic-associated steatotic liver disease, *OR* odds ratio, *PAD* peripheral artery disease, and *RR* relative risk See reference [5] for full reference information of studies mentioned in the table. (Reprinted with permission from: Arterioscler Thromb Vasc Biol. 2022;42:e168-e185. ©2022 American Heart Association, Inc. [5].)				

Although these data suggest an association between liver fat and CVD risk, this relationship has been called into question. A large meta-analysis involving greater than 498,000 subjects across fourteen studies demonstrated that those with MASLD were at an increased risk of death from all causes compared to those without (HR = 1.34; 95% CI 1.17–1.54) [32] but there was no association between MASLD and CVD-specific mortality (HR = 1.13; 95% CI 0.92–1.38) [32]. The authors argued that the association between MASLD and all-cause mortality may be attributable to liver-related mortality (HR = 2.53; 95% CI 1.23–5.18) rather than CVD mortality. Important limitations of this meta-analysis include significant heterogeneity with regards to the association between MASLD and all-cause mortality (I^2^ = 80.0%, *P* < 0.01) and moderate heterogeneity in the analysis involving CVD mortality (I^2^ = 57.5%, *P* = 0.03).

An explanation for the discordant results amongst available studies investigating the association between MASLD and CVD may lie in the effect of metabolic parameters that serve as confounders closely linked to both MASLD and CVD and difficulty separating these. This was highlighted by a recent multicohort analysis of greater than 10,000 participants from the Framingham Heart Study, the Coronary Artery Risk Development in Young Adults Study, and the Multi-ethnic Study of Atherosclerosis investigating the relationship between liver fat and incident CVD longitudinally using both baseline and time-varying covariates. Higher liver fat was associated with incident CVD (HR: 1.08; 95% CI 1.01–1.14) in the multivariable model using baseline covariates excluding BMI [33]. When BMI was included as a covariate, a significant association was no longer present (HR 1.04; 95% CI 0.97–1.11) [33]. When time-varying covariates instead of baseline covariates were used, liver fat was no longer significantly associated with incident CVD regardless of the inclusion of BMI as a covariate (without BMI HR 1.06; 95% CI 0.98–1.14; with BMI HR 1.02; 95% CI 0.95–1.11) [33]. These data suggest that rather than acting as an independent risk factor for CVD, liver fat may appear to be associated with CVD due to its close association with other well-established risk factors for CVD, such as BMI. These data demonstrate how accounting for changes in CVD risk factors over time is crucial for understanding the above relationship.

The complexity of the relationship between hepatic fat and CVD is further highlighted by data suggesting hepatic fat may be associated with a lower incidence of ASCVD events in those with T2D. An analysis of over 1,200 Dallas Heart Study (DHS) participants investigating the association between hepatic triglyceride content (HTC) and ASCVD events after stratification for T2D status revealed an inverse association between HTC and ASCVD events in those with T2D (HR 0.90; 95% CI 0.82–0.98) [34]. Importantly, no relationship was present in those without T2D [34]. Furthermore, this inverse relationship between HTC and ASCVD events, limited to coronary heart disease, in those with T2D was replicated in an analysis of over 37,000 participants from the UK Biobank (UKB) (HR 0.95; 95% CI 0.90–0.99) [34].

In addition to T2D status, the balance of fat depots appears to be of significant importance when considering CVD risk. In a study combining data from DHS and UKB, an obesity phenotype with high VAT combined with low liver fat was most strongly linked to CVD incidence, while T2D was most strongly associated with an obesity phenotype featuring high VAT and high liver fat [35]. Similar results were demonstrated in an analysis of over 40,000 participants from the UKB in which body fat z-scores were calculated to characterize specific fat depot size relative to those with similar BMI. Again, excessive VAT in relation to BMI was most strongly associated with both CVD and T2D and excessive liver fat in relation to BMI was not associated with CVD risk after adjusting for age and sex [36].

Given the abundance of discordant results regarding the association between liver fat and CVD outcomes, high quality evidence free of confounders, such as a randomized control trial, are necessary. Given ethical and practicality concerns, this is unlikely to occur. With the advent large genetic databases and genome-wide association studies, Mendelian randomization (MR) approaches have been developed to address questions not amenable to randomized control trials. In a 2022 MR study, Peng et al. utilized the Million Veteran Program (MVP) to identify single nucleotide polymorphisms (SNPs) associated with MASLD [37]. Strict selection criteria were used to identify SNPs to be utilized as instrumental variables. Outcome data regarding arterial stiffness, heart failure, coronary artery disease, and stroke were obtained from publicly available databases and a two-sample MR analysis was performed to expose the potential causal effect of MASLD on these outcomes. Although there was a significant association between genetic-predictors determined MASLD and arterial stiffness (β = 0.04; 95% CI 0.02–0.06), there was no association with CAD or any stroke subtype [37]. While arterial stiffness can be a marker of subclinical atherosclerosis, the absence of an association with clinical CAD or any subtype of stroke supports the theory that MASLD may not be an independent risk factor for CVD beyond its relationship with traditional CV risk factors.

### Interventions

Currently, there are three principal modalities by which obesity is managed including lifestyle/behavioral modifications, pharmacologic therapy, and surgical intervention. The goal of intervention is to decrease morbidity and mortality associated with obesity by addressing associated complications and to improve metabolic parameters including lipid profile, serum glucose levels, insulin sensitivity, and body composition. Historically, surgical management has been associated with superior long term outcomes for weight reduction and correction of metabolic imbalances; however, emerging evidence and recent pharmacologic advances have the potential to redefine the landscape of personalized medicine and obesity management [38, 39].

#### Lifestyle Modifications

Comprehensive lifestyle modifications, including a combination of diet/exercise regimen and behavioral modifications, remains the initial approach for the management of obesity. Several dietary regimens including caloric restriction (CR) and the Mediterranean diet have been associated with weight, hepatic fat content, and waist circumference reduction and improvement in dyslipidemia, glycemic control, and insulin sensitivity [40, 41]. Recently gaining popularity, intermittent fasting (IF) improves body weight/composition and cardiometabolic health risk factors as compared to unrestricted dieting; however, further trials are needed to elucidate if IF provides further health benefits as compared to CR [42]. Additionally, programs incorporating meal replacement nutritional offerings (as either part of total diet replacement program or taken daily in place of snacks/meals) have led to increased weight loss as compared to programs without meal replacement options with significantly reduced cardiovascular risk factors [43, 44].

Physical activity is an essential component to lifestyle modification and should be encouraged for all patients as its role in reducing mortality and CVD risk is well described [45]. The benefit of exercise on cardiovascular health is independent on concurrent weight loss, as demonstrated by the EPIC-Norfolk study that showed exercise was associated with decreased risk of coronary heart disease in patients with metabolic syndrome (MS) even in those with stable weights [46]. Additionally, a recent meta-analysis revealed that exercise interventions resulted in greater VAT reduction relative to weight loss as compared to pharmacologic interventions suggesting that measuring the benefit of exercise with weight loss alone underestimates its impacts on cardiovascular health [47]. Recent studies have shown that cardiovascular fitness (CRF), rather than weight loss alone, influences the relationship between obesity and mortality in those with established CVD [48]. CRF is increasingly being recognized as a risk modifier in both healthy individuals and in persons with cardiometabolic comorbidities, particularly in heart failure. Notably, when CRF is accounted for, the obesity paradox disappears, suggesting that improving CRF might represent a therapeutic target with greater importance than changes in body weight in the setting of heart failure [49].

Despite the clear benefit of lifestyle modification on body weight, waist circumference, visceral/hepatic fat content, cardiovascular health, and cardiometabolic risk profile, the efficacy of managing obesity with lifestyle modifications alone is limited by maintenance and adherence; and such interventions have been unable to demonstrate sustained risk reduction in patients with severe obesity [8]. As such, lifestyle modifications are often used alongside pharmacologic and/or surgical interventions that are associated with more sustained weight loss and improved outcomes [50].

#### Pharmacologic

There are numerous pharmacologic agents used in the management of obesity; and pharmacotherapy used alongside lifestyle medications have been shown to produce greater weight loss than simply implementing lifestyle changes alone (Table 2) [51, 52]. Recent research into sodium glucose co-transporter 2 inhibitors (SGLT2i) and glucagon-like peptide – 1 (GLP-1) receptor agonists have revealed significant impacts on body fat distribution and have the potential to redefine the medical management of obesity.

**Table 2:** Weight loss effects of FDA approved anti-obesity medications

Medication	Route and Frequency	Weight Loss Efficacy	Adverse Effects	Contraindications
Orlistat (Xenical/Alli)	Oral 3 times daily	≥ 5%, 21% ≥10%, 12%	Diarrhea, oily stools Fecal incontinence Fat soluble vitamin deficiency	Pregnancy Chronic malabsorption Cholestasis
Phentermine/ topiramate ER (Qsymia)	Oral Daily	≥ 5%, 67% ≥ 10%, 47%	Headache, dizziness, fatigue Nausea, dry mouth, constipation Hypoglycemia, back pain, cough	Pregnancy Glaucoma, hyperthyroidism Uncontrolled hypertension
Naltrexone/ bupropion ER (Contrave)	Oral Twice daily	≥ 5%, 42–57% ≥ 10%,21-35%	Headache, dizziness, insomnia Nausea, dry mouth Constipation, diarrhea	Pregnancy Uncontrolled hypertension, seizure disorder opioid use, eating disorder, monoamine oxidase inhibitor use
Liraglutide (Saxenda)	Subcutaneous Daily	≥ 5%, 62% ≥ 10%, 34%	Nausea/vomiting Diarrhea, constipation Headache, dizziness, fatigue Hypoglycemia, abdominal pain	Pregnancy Personal/family history of medullary thyroid cancer Multiple endocrine neoplasia type 2, caution in history of pancreatitis
Semaglutide (Wegovy)	Subcutaneous Weekly	≥ 5%, 86% ≥ 10%, 69% ≥ 15%, 50%		
Tirzepatide (Zepbound)		≥ 5%, 91% ≥ 10%, 83% ≥ 15%, 71%		

SGLT2 inhibitors exert their metabolic effects primarily through coupling of natriuresis with osmotic diuresis via blood glucose dependent glycosuria and urinary sodium excretion with subsequent reduction in blood sugars and blood pressures [53]. SGLT2 inhibitors have been associated with cardiovascular, renal, and hepatic protective benefits. Specifically, clinical trials including DAPA-HF, EMPEROR, CREDENCE, DAPA-CKD, and EMPA-KIDNEY have established SGLT2 inhibitors protective effects as evidenced by the reduction of worsening heart failure and progression to kidney failure in patients with heart failure and/or chronic kidney disease (CKD). Further studies have shown that these protective effects of SGLT2i are similar in patients with or without diabetes [54]. A recently published study further postulates that the mechanism of the hepatoprotective effect of empagliflozin is perhaps related to counteraction of hepatic gluconeogenesis, improved hepatic mitochondrial functions, and beneficial effect against hepatic inflammation and fibrosis [55].

Additionally, SGLT2 inhibitors not only result in modest weight loss, but this class of medication has also been reported to impact adipose tissue distribution and body composition. A recent meta-analysis provided supporting evidence that in patients with T2D, SGLT2 inhibitors can significantly reduce VAT, subcutaneous adipose tissue, and ectopic hepatic fat [56]. Furthermore, several studies support that SGLT2 inhibitors promote reduction of VAT in patients with T2D, improve hepatic steatosis with reduction in liver fat content in patients with T2D and MASLD, and support positive effects on cardiometabolic biomarkers in patients with T2D suggesting that early administration of SGLT2 inhibitors in patients with T2D and MASLD may be favorable [57–63]. Lastly, SGLT2 inhibitors have also been found to reduce the incidence of obstructive sleep apnea and have favorable impact on risk factors for cardiovascular outcomes in patients with T2D and ASCVD [64, 65].

GLP-1 receptor agonists impact weight loss and glycemic control through several mechanisms, most notably via glucose dependent stimulation of insulin release, delaying gastric emptying, inhibition of glucagon secretion, and appetite suppression with increased satiety [8, 66]. The benefit of GLP-1 RA in the management of visceral obesity has been well studied in patients with underlying T2D and these medications have been shown to significantly reduce visceral fat and hepatic fat content with improved glycemic control in this population [67–69]. More recent literature suggests that these benefits are also seen in patients with obesity without T2D. Specifically, liraglutide in combination with lifestyle modifications has been shown to significantly reduce visceral and ectopic fat \and adverse muscle composition as compared to placebo in overweight or obese adults without diabetes. Furthermore, the combination of liraglutide and adherent exercise has been shown to reduce metabolic syndrome severity, abdominal obesity, and the proinflammatory state associated with metabolic syndrome in this population [70–72]. Of particular clinical significance, recent landmark studies have also attributed this class of medication to reduction in the incidence of MACE in populations with T2D and those with obesity as evidenced by the LEADER and SELECT trials respectively [73, 74]. Even more recently, tirzepatide, a dual glucose dependent insulinotropic polypeptide and GLP-1 RA, is undergoing evaluation in the SURPASS trial series. Current data suggests tirzepatide has led to greater reductions in HbA1C, body weight, liver fat content, visceral adipose tissues, and abdominal subcutaneous adipose tissues as compared to insulin degludec in patients with T2D [75–77].

Despite exciting research and pharmacologic breakthroughs surrounding the medical management of obesity, further studies are needed to evaluate translating initial weight loss and improvement in metabolic parameters with correlation to long term clinical benefit and sustained weight loss.

#### Surgical

Surgical management of obesity is indicated in adults with BMI ≥ 30 kg/m^2^ and at least one major obesity related complication (e.g., diabetes, hypertension, hyperlipidemia, coronary artery disease, OSA, and others) or in patients with BMI ≥ 35 kg/m^2^ without obesity related disease [66, 78]. As previously mentioned, surgical management is associated with superior long-term efficacy as compared to medical management. More specifically, a recent meta-analysis revealed that bariatric surgery demonstrated statistically significant superiority in provided weight loss, total cholesterol, triglycerides, systolic and diastolic blood pressure, and cardiovascular risk [79]. Similarly, bariatric surgical techniques have also been associated with more profound impact on overall body composition as compared to medical therapy; particularly with proportionally more VAT reduction as compared to lean mass reduction [80]. Lastly, weight loss and visceral fat reduction following bariatric surgery has been associated with favorable cardiac effects including reduction in epicardial fat, LV reverse remodeling, and improved longitudinal biventricular mechanics suggesting that VAT loss should be further investigated as a primary driver of improved cardiac function [1]. The highlighted benefits of surgical management of obesity reflect the importance of identifying patients who would be more appropriately managed with aggressive surgical management as compared to medical management.

## Conclusions

Although obesity has an important association with cardiometabolic health, fat depots, including VAT, SAT, and hepatic fat, are important metrics that are not adequately captured by BMI alone. Clear associations between VAT and various cardiometabolic outcomes, including CVD, T2D, and OSA have been demonstrated. Meanwhile, the association between hepatic fat and cardiometabolic outcomes remains less clear. Although comprehensive lifestyle management remains the initial approach for the management of obesity, safe and effective pharmacotherapy supported by robust cardiometabolic outcomes data have emerged.

## Key References

- Ndumele CE, Rangaswami J, Chow SL, Neeland IJ, Tuttle KR, Khan SS, et al. Cardiovascular-Kidney-Metabolic Health: A Presidential Advisory From the American Heart Association. Circulation. 2023;148:1606–35.
  - This Scientific Statement Describes the inter-related Pathophysiology and Clinical Impact of Cardiovascular, Kidney, and Metabolic Disease and its Direct Relation to Excess Visceral and Ectopic fat.

- Tejani S, McCoy C, Ayers CR, Powell-Wiley TM, Després J-P, Linge J, et al. Cardiometabolic Health Outcomes Associated with Discordant Visceral and Liver Fat Phenotypes: Insights from The Dallas Heart Study and UK Biobank. Mayo Clin Proc. 2022;97:225–37.
  - Findings from this study suggest heterogeneity in the relationships of visceral and liver fat with cardiometabolic outcomes.

- Neeland IJ, Marso SP, Ayers CR, Lewis B, Oslica R, Francis W, et al. Effects of liraglutide on visceral and ectopic fat in adults with overweight and obesity at high cardiovascular risk: a randomised, double-blind, placebo-controlled, clinical trial. The Lancet Diabetes & Endocrinology. 2021;9:595–605.
  - Findings from this study demonstrate the effects of the GLP-1 RA, liraglutide, on visceral and ectopic fat in persons with overweight and obesity.

## Data Availability

No datasets were generated or analysed during the current study.

## Abbreviations

ASCVD: atherosclerotic cardiovascular disease
BMI: body mass index
CAD: coronary artery disease
CKM: cardiovascular–kidney–metabolic
CR: calorie restriction
CVD: cardiovascular disease
DHS: Dallas Heart Study
GLP1: glucagon–like peptide 1
HTC: hepatic triglyceride content
IF: intermittent fasting
MACE: major adverse cardiovascular events
MASLD: Metabolic dysfunction associated steatotic liver disease
MI: myocardial infarction
MR: Mendelian Randomization
MS: metabolic syndrome
MVP: Millio Veteran Program
NHANES: National Health and Nutrition Examination Survey
OSA: obstructive sleep apnea
SAT: subcutaneous adipose tissue
SGLT2: sodium–glucose transporter 2
SNPs: single nucleotide polymorphisms
T2D: type 2 diabetes
UKB: UK Biobank
VAI: visceral adiposity index
VAT: visceral adipose tissue
